# Effects of Stroking on Salivary Oxytocin and Cortisol in Guide Dogs: Preliminary Results

**DOI:** 10.3390/ani10040708

**Published:** 2020-04-18

**Authors:** Asahi Ogi, Chiara Mariti, Paolo Baragli, Valeria Sergi, Angelo Gazzano

**Affiliations:** Department of Veterinary Sciences, University of Pisa, Viale delle Piagge 2, 56124 Pisa, Italy; chiara.mariti@unipi.it (C.M.); paolo.baragli@unipi.it (P.B.); v.sergi@studenti.unipi.it (V.S.); angelo.gazzano@unipi.it (A.G.)

**Keywords:** oxytocin, saliva, dog, isolation, human–animal interaction, cortisol

## Abstract

**Simple Summary:**

Oxytocin, a nonapeptide hormone produced by the hypothalamus and secreted by the pituitary gland, plays a peripheral role during labor, birth, and nursing. Since this role is well known nowadays, scientific interest in this hormone has shifted from its peripheral functions to its central functions, such as the onset of parental care, the regulation of social bonding, and modulation of the emotional state. For this final reason, this study intended to investigate the possible variations of salivary oxytocin induced by two different conditions: a positive condition (5 min of human–dog interaction) and a negative condition (5 min of isolation). Because oxytocin function is known to be affected by stress, salivary cortisol concentration was determined after both conditions and a stress–behavior assessment during the isolation phase was performed to further assess signs of distress. A significant increase in salivary oxytocin was observed only during the positive condition and, although no signs of distress were observed during the isolation phase, a correlation between the variations of salivary oxytocin and cortisol concentrations was found. These findings highlight the possibility of measuring the potential impact of stress on the oxytocinergic system during behavioral tests.

**Abstract:**

This pilot study aimed at investigating how salivary oxytocin levels are affected by human interaction and isolation in eight guide dogs (six Labrador retrievers and two golden retrievers; four males and four females, 21.87 ± 1.36 months old) just before assignment to the blind person. Each dog engaged, at one-week intervals, in a positive (5 min of affiliative interaction with their trainer) and a negative (5 min of isolation) condition. Saliva samples used for Enzyme Immunoassay (EIA) quantification of salivary oxytocin were collected before and immediately after both experimental conditions. In order to assess potential hypothalamic pituitary adrenal (HPA) axis activation that could have affected oxytocin levels, saliva samples were collected 15 min after both experimental conditions for EIA quantification of salivary cortisol and a behavioral assessment was performed during the negative condition. The results were compared using the Wilcoxon test (*p* < 0.05). Oxytocin concentrations showed a statistically significant increase after the positive interaction (*p* = 0.036) and no difference after the negative one (*p* = 0.779). Moreover, no difference (*p* = 0.263) was found between the cortisol concentrations after each experimental condition and no signs of distress were observed during the isolation phase. These preliminary findings support the hypothesis that stroking dogs has positive effects on their emotional state independently of hypothalamic pituitary adrenal axis activation.

## 1. Introduction

Oxytocin (OXT) is a peptide of nine amino acids hormone produced by the hypothalamus and secreted by the pituitary gland. Its name, derived from the Greek words for “quick birth,” evokes its essential role in parturition, but this hormone also plays a central role in general behavioral regulation, particularly in positive social interactions [1]. These aspects have been intensively investigated in recent years in different animal species.

Since the 1990s, the prairie vole (*Microtus ochrogaster*), a monogamous rodent found in central North America, has been used to study the role of OXT in social behavior. In 1990 it was proven that intracerebroventricular injections of oxytocin in female prairie voles were able to facilitate social contact [2] and, using the same procedures with different dosages, in 1991 Mahalati et al. showed that OXT produced an immediate cessation in sexual behavior in sexually-active male prairie voles. In 1992, Insel et al. [3] found contrasting patterns of oxytocin receptor expression in monogamous and polygamous voles’ brains; finally, in 1994, Williams et al. [4] showed that OXT can facilitate the onset of selective partner preferences. Over the years, the “quick birth” hormone was investigated in many other studies on this model’s sociosexual behavior [5,6], maternal and affiliative behavior [7,8], stress-coping behavior [9], social isolation [10], and social attachment [11].

Regarding social behavior, OXT secretion after positive social interaction is widely observed in primates [12,13,14,15], but the impact of relationship strength on OXT release is not yet well known. In one study on chimpanzees, urinary OXT concentration increased after grooming with a bonded partner, but not after the same interaction with a nonbonded partner [13]; on the contrary, two studies suggest that OXT secretion is independent of affiliative relationship strength [14,16].

In humans, in addition to the study on social behavior, oxytocin has been linked to autism spectrum disorder (ASD) [17] and its potential treatment [18].

In dogs, OXT has become a biomarker commonly investigated in behavioral and neuroendocrinological research, but, in contrast to the investigation on serotoninergic system, commonly associated with aggressive behavior [19], the oxytocinergic system has been frequently linked to sociopositive human–dog interaction [20]. Actually, the behavioral effects of oxytocin in dogs are mainly based on three macro-areas: the effect of human–dog social interaction on peripheral oxytocin levels, the associations between the polymorphisms in oxytocin receptor gene and social behavior, and the effect of exogenous oxytocin administration on dogs’ social behavior [21]. Dogs’ positive social interaction with humans has mostly been investigated in companion dogs living with their owners [22,23,24,25]. Usually, dogs are a nonhomogeneous sample because they have different backgrounds and, in most cases, different ages and breeds. A good uniformity of samples can be found in the studies of Handlin et al. [26,27] (10 male Labradors but of different ages and backgrounds), Rhen et al. [28] (12 laboratory-kept beagles of about the same age), and MacLean et al. [29] (11 Labrador retrievers and 27 Labrador retriever × golden retriever crosses of roughly the same age, living conditions, and training).

OXT concentrations can be peripherally quantified in the plasma, urine, and saliva. Blood sampling is an invasive medical procedure that could cause some stress and pain; moreover, blood OXT concentrations can vary, even within 90 s of the onset of a stimulus [30], and may be measured wrongly due to bonding with other molecules [31]. Urine sampling is a noninvasive procedure, but it has the problem of timing, especially for testing the effect of short-term stimuli, and the significance of determining urinary OXT concentration is still unclear [32]. Lastly, salivary OXT sampling has none of these disadvantages; it is a noninvasive procedure, it can be performed with specific and accurate timing (although the right timing of OXT peak in saliva has not yet been accurately established), and it can be done directly by owners in the dogs’ usual environment. Furthermore, salivary EIA measurement can also be carried out without a solid-phase extraction [33].

Contrary to what was observed in lambs, in which plasma OXT levels increased during isolation and decreased during an interaction with a familiar caregiver [34], recent studies suggest that OXT facilitates and increases after affiliative, positive forms of human–animal interaction, including in domestic dogs [22,26,29]. From a neurophysiological point of view, the secretion of OXT has inhibitory actions on the activity of the hypothalamic–pituitary–adrenal (HPA) axis [35]; additionally, in response to a stressor, OXT is released in the paraventricular nucleus (PVN), which is subsequently associated with active stress-coping behavior [36]. This mechanism seems to be confirmed in humans. In fact, a study on the link between romantic attachment anxiety and levels of plasma OXT supports the hypothesis that oxytocin levels may rise in response to relationship anxiety [37]. Another study in humans found that higher blood OXT levels after a stressful interpersonal task were associated with more anxiety, suggesting that oxytocin could be a marker of distress [38]. Furthermore, in humans oxytocin levels seem to be positively correlated with increased maternal separation distress in children [39].

Based on previous studies in humans, oxytocin and cortisol concentrations are two variables that are inversely proportional during, and after, a positive interaction (“non-noxious sensory stimulation” [26]) and, apparently, also at baseline [40]. Therefore, a decrease in plasma cortisol concentrations might be expected in response to a positive experience in dogs as well, but two recent studies showed an increase in cortisol levels after a positive human–animal interaction set up by non-noxious sensory stimulation [22,26]. A possible explanation of this unexpected result could be the influence of a new environment, which could be stressful, or stimulating, for dogs [22,41]. However, the possible influence of a new environment during behavioral tests on dogs’ emotional and hormonal response has not yet been well investigated. Another possible explanation of the increase in cortisol levels could be the hormonal synchronization between humans and dogs [42].

Consistently with the existing literature, we hypothesized that a non-noxious sensory stimulation, consisting of positive short-term human–dog interaction, could lead to an increase in OXT levels in dogs. Moreover, unlike the previous studies, to deeper investigate the possible role of the environment and hormonal synchronization on HPA axis activation, we assessed the physiological response to an unfamiliar test room during social isolation, without the presence of any other sensorial stimulus. Finally, to prevent possible bias resulting from different backgrounds of the subjects, this pilot study was performed on eight trained guide dogs just before assignment to the new, blind owners.

## 2. Materials and Methods

The study was approved by the Ethical Committee of the University of Pisa, Italy (protocol # 63/2018) in accordance with Directive 2010/63/EU.

Eight guide dogs from the Guide Dogs National Training School, located in Scandicci (Florence, Italy) were included. Six Labrador retrievers and two golden retrievers, four of them castrated males and four of them spayed females (mean ± standard deviation age = 21.87 ± 1.36 months), which had concluded their training, were recruited. The dogs were all healthy and highly socialized.

The training of the dogs is completely standardized according to the guidelines and standards of the International Guide Dog Federation. Between two and 14 months of age, the dogs live with preselected families; every month between two and five months of age, the puppies take part, with their caregivers, in a puppy class with their brothers and sisters or preselected puppies of the same age; between five and 14 months of age they spend an entire week at the school every month; at the age of 12 months the dogs are behaviorally tested and, two months later, only the suitable dogs start the actual six-month training. Each dog spends 40–50 min per day working with its assigned trainer and each dog is behaviorally tested twice during the training period, once in the middle and a second time at the end. Finally, they spent two weeks at the school with the blind person before their assignment.

Four trainers, three women (trainers A, C, and D) and one man (trainer B), with comparable experience, were recruited. Trainer A trained dogs 1 and 2, trainer B dogs 3 and 4, trainer C dogs 5 and 6, and trainer D dogs 7 and 8.

The study was carried out two weeks before the assignment of the dogs to the new, blind owners. It consisted of exposing the dog to a new physical environment during a positive condition (PC, 5 min of affiliative interaction with their trainer, who had been instructed to stroke and speak gently to the dogs) and during a negative condition (NC, 5 min of isolation). Each condition took place in a room unfamiliar to the dogs; in order to maintain unfamiliarity, two rooms were used, one for each test, as similar as possible to each other—the same square footage (3 × 3 m) and same layout. The tests were performed at a one-week interval, and the order of the two different conditions and the use of the two different rooms were randomly selected. Before each 5-min condition, the dogs had 1 min to explore the environment with their trainers, in order to reduce their arousal for the new environment and mitigate HPA axis activation.

Saliva samples were collected by the trainers, supervised by the experimenter, before (T0), immediately after (T1), and 15 min after (T2) both experimental conditions. Salivette^®^ (Sarstedt, Rommelsdorft, Germany) was used for collecting the samples. The swabs were gently put under the tongue and in the cheek pouches of the dogs for 60 s. Before the tests, each dog was left in a single, familiar, indoor, and quiet crate (length × width × height = 122 × 77 × 82 cm) for 30 min. Basal samples of saliva were collected directly inside the crate (T0); these samples were used to measure only OXT levels because the amount of the saliva samples was not enough to also measure the cortisol concentration. Following the first/basal sample, the dogs remained for a further 15 min in the same crate and then were led by their trainers into one of the unfamiliar test rooms. Immediately after the two experiments (T1), another saliva sample was collected in the test room by the trainer for the measurement of OXT levels. Finally, the dogs were led back to their crates for an additional 15 min, when the last samples were collected (T2), in accordance with the timings previously reported for determining salivary cortisol concentrations in dogs after exposure to a stressor [43,44]. All samples were immediately refrigerated and brought to the Etovet laboratory of the Department of Veterinary Sciences—University of Pisa (Italy) for centrifugation and stocking at −20 °C, until they were analyzed for oxytocin quantification with a Cayman Chemical ELISA Kit^®^ (Item #500440) (Ann Arbor, MI, USA), previously validated for use in dogs [33], and cortisol quantification by Diametra Cortisol Enzyme Immunoassay Kit^®^ (Spello, PG, Italy), according to the manufacturer’s instructions.

The behavior of the dogs was video recorded with a Sony^®^ HDR-CX190E camera (Sony Corporation, Kōnan, Minato, Tokyo, Japan) during the 5-min isolation phase (NC). The camera was positioned on a 150-cm-tall tripod located laterally to the door of the room. To prevent the dogs from getting too close to the camera, a puppy playpen was placed around the tripod. Each video was analyzed through a continuous sampling method with BORIS^®^ v. 6 [45], following a specific ethogram of stress-related and nonstress-related (lying, sitting, standing, and exploration) behaviors (Table 1). According to the BORIS^®^ guidelines, two types of behaviors were defined: point event (PE) behavior, when the number of occurrences were analyzed, and state event (SE) behavior, when both the duration and the number of occurrences was measured.

The Wilcoxon test (*p* < 0.05) was used to assess possible differences between the oxytocin and cortisol concentrations at different times and in both conditions. The Shapiro–Wilk test was used to determine the skewness of distribution, and Spearman’s rank correlation coefficient (rho) was used to assess possible correlations between salivary cortisol and oxytocin concentrations, and between stress-related behaviors and both analyzed hormones. The statistical analysis was performed through IBM SPSS^®^ (Statistical Package for the Social Sciences) v. 17 (Armonk, NY, USA).

## 3. Results

No significant difference was found between basal salivary oxytocin concentrations (T0) in the two different experimental conditions (*p* = 0.401), and no significant difference was found between male and female salivary concentrations of oxytocin at T0 (*p* = 0.207). The starting conditions were therefore considered equal and the group was considered homogeneous.

**Figure 1:**
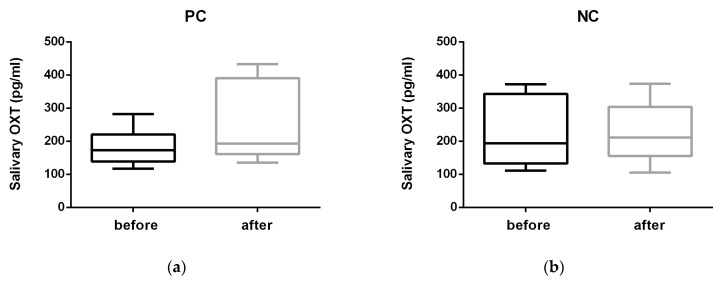
Salivary OXT (oxytocin) concentration (pg/mL) before and after (**a**) the positive condition consisting of 5 min of affiliative interaction between dogs and their trainers (PC) and (**b**) the negative condition consisting of 5 min of isolation (NC).

Oxytocin concentrations (Figure 1) showed a statistically significant increase immediately (T1) after the PC (before versus after: median 172.77 versus 193.77 pg/mL, min–max 117.97–282.23 versus 135.80–433.11 pg/mL; Z = −2.100; *p* = 0.036) and no difference after the NC (193.97 versus 211.35 pg/mL, 111.10–372.10 versus 105.30–374.10 pg/mL; Z = −0.280; *p* = 0.779).

No difference (*p* = 0.263) between cortisol concentrations was found after either condition (PC: mean ± standard error of the mean (SEM) = 0.18 ± 0.03 µg/dL, NC: mean ± SEM = 0.14 ± 0.02). Furthermore, no correlation between all observed behaviors (Table 2) and either salivary cortisol or ΔOXT (oxytocin levels at T1–oxytocin levels at T0) was observed during the negative condition.

In both conditions, no correlations between the gender of the four trainers and the hormonal response in dogs were observed.

Finally, there was no correlation between salivary cortisol and oxytocin concentrations, but a positive correlation was found between ΔOXT and cortisol concentrations (rho = 0.810) during the isolation phase (Table 3).

## 4. Discussion

Regarding the positive condition by itself, these preliminary findings basically confirm that stroking dogs generates a significant increase in their salivary oxytocin concentration [29]. Comparing the subjects tested by McLean et al. [29] with our subjects, we observed many similarities—they were about the same age, of the same breed, and had the same living conditions and training target. However, while they opted to split their subjects into two groups, we decided to test each dog for the PC and NC at a one-week interval. As far as our group of dogs could be considered emotionally and physically homogeneous, it is noted that the limited number of subjects could have led to statistical bias and a misinterpretation of the data.

Regarding the isolation phase and the comparison between the two conditions, a deeper investigation must be done.

Because social isolation might be one of the causes of the increase in stress-related behaviors [58] and therefore of the increase in cortisol and oxytocin concentrations [36], it should be noted that these dogs are used to being left alone; in fact, the behavioral analysis showed no significant signs of distress or locomotory activation. In this regard, it can be said that the dogs spent more than 50% of the time lying on the ground and showed limited distress behaviors, such as licking, yawning, and vocalization, and they were oriented to their environment [59]. In fact, although their attention was oriented to the door for 46% of the time, basically no behaviors oriented to the door were recorded. The same can be said about the locomotory activity. In addition, to confirm this hypothesis, the comparison of salivary cortisol concentrations at T2 showed no statistically significant difference between the two conditions and the salivary cortisol levels were all in the basal range, according to a systematic review of salivary cortisol in dogs [60].

It has been suggested that petting may reduce the stress response and increase relaxation in dogs, due to the oxytocin release [43,61]; even if no statistically significant differences in cortisol levels were found between the two conditions, these preliminary results suggest that stroking promotes a significant increase in oxytocin compared to an unstressful situation. In contrast with the existing literature [22,26,62], the increase in oxytocin during the positive PC was not followed by any significant change in cortisol levels compared to the NC. These discrepancies could be due to the characteristics of the subjects. Handlin et al. tested 10 privately-owned male Labradors older than one year but of different ages and backgrounds [26,27]; Odendaal and Meintjes tested 18 privately-owned dogs of different breeds and between two and 11 years of age [22]. Another reason for these different outcomes could be the different sampling methods, because they determined cortisol levels using blood samples [22,26,27].

However, the cortisol concentration after the PC (mean ± SEM = 0.18 ± 0.03 µg/dL) was higher compared to the NC (mean ± SEM = 0.14 ± 0.02), maybe due to the positive arousal [63]. The comparison with a negative, but static and unstressful, situation prevented possible bias due to the endogenous release of oxytocin in response to distress, as reported in humans [35,36,37,38,39], or due to locomotory activation [26]. In contrast to the studies mentioned above, we hypothesize that a decrease in salivary OXT could be correlated with a negative emotional impact. Exclusively considering the outlier, dog number 8, we observed the greatest ΔOXT (−162.19 pg/mL) and the highest duration of whining/yelping (57.7%). In this specific case, we cannot be confident that the isolation did not affect the salivary OXT level.

Moreover, the isolation prevented a possible hormonal activation, even considering a possible social and stress synchrony in the handler–dog dyad, as suggested by Pirrone et al. [64].

Unlike the results found during a dog agility competition by Buttner et al. [42], and although previous studies have found that men and women interact with dogs differently [65,66], we did not observe a difference in the cortisol levels and ΔOXT of dogs belonging to males compared to those of female trainers, probably because in our study the interactions were very short and standardized. However, considering the limited and non-gender-balanced number of trainers, our results should be considered with caution.

Regarding the correct timing of saliva samples, a study conducted by Handlin et al. [26] on short-term interactions between dogs and their owners reported that the dogs’ peak oxytocin levels recorded after just 1 min of interaction were significantly higher than the levels collected at time point 0 min [26], suggesting that the peak of blood OXT is immediately after the onset of a positive stimulus. Even though a study conducted in young men reported no correlation between blood and salivary OXT [67], others reported the opposite [68,69,70]. It follows that, if the hormone release in the peripheral circulation is immediate, the increase in saliva is probably at least rapid, although the time lag between blood and saliva peaks of this hormone could be slightly different depending on the species, as with cortisol [71]. To confirm the hypothesis of a rapid release of salivary OXT in dogs, our study reported a significant increase in saliva levels immediately after 5 min of positive interaction, as also recently shown by MacLean et al. [29].

Although there are some studies suggesting the correct timing to collect saliva samples to assess OXT concentration peaks in dogs, there is no consistency on this topic and, to the best of the authors’ knowledge, there is no certainty about the specific timing and duration of salivary OXT peaks in dogs. This consideration may have been a bias and needs to be considered when assessing variations in OXT concentration in response to stimuli.

The positive correlation between the difference in oxytocin levels at T1 and T0, and the cortisol concentration during the isolation phase, might suggest a possible influence of oxytocin variation on the HPA axis or vice versa, even though no direct correlation was found between salivary oxytocin and cortisol. The inhibitory action of oxytocin on the activity of the HPA axis [35] could have limited the increase in cortisol levels. This could mean that a negative, but unstressful, stimulus could be identified only by a difference between salivary oxytocin measured before and after the stimulus itself. However, these results are not adequate to assert that ΔOXT could be considered an early marker of stress; future research, with a larger number of dogs and with a higher level of stress, is needed for a better understanding of the specific factors that affect the correlation between cortisol and oxytocin.

## 5. Conclusions

Our preliminary findings confirm that stroking dogs generates a significant increase in their salivary oxytocin concentration compared to a negative but unstressful situation. Moreover, as suggested by Handlin et al. [26], in the NC we avoided the possible influence of the owner’s emotional state on hormonal response of the dogs.

As far as the HPA axis activation, our results need to be interpreted with caution. Even if we did not observe a significant difference between PC and NC, the higher cortisol level recorded during the PC could be indicative of positive arousal [63] or stress synchrony [42,64]. Moreover, the inhibitory action of the oxytocin on the activity of the HPA axis [35] could have limited the increase in cortisol levels during the NC.

One of the strengths of our study is the uniformity of the participating dogs, in terms of both emotional and physical characteristics. This kind of sample and the use of dogs as their own control ensured a reduction of the risk of bias due to a small number of dogs. For this reason, the present pilot study may help clarify some timing aspects of collecting saliva samples and highlights the possible impact of HPA axis activation on the oxytocinergic system during behavioral tests.

Most importantly, this study suggested, through an increase in salivary oxytocin levels, that gentle touching is beneficial for dogs not only while experiencing or after having experienced a stressful event [43,72], but also during a neutral situation.

## Figures and Tables

**Table 1:** Ethogram.

ETHOGRAM		
Behavior	Definition	References
Lying (SE)	Ventral/lateral lying on ground with all four legs resting and in contact with ground	[46]
Sitting (SE)	Hindquarters on ground with front two legs being used for support	[46,47]
Standing (SE)	All four paws on ground and legs upright and extended, supporting body	[46,47]
Exploration (SE)	Activity directed towards physical aspects of the environment, including sniffing, close visual inspection, distal visual inspection, and gentle oral examination, such as licking	[48,49,50]
Locomotion (SE)	Walking, running, pacing around without exploring the environment or playing	[46]
Attention oriented to the door (SE)	Staring fixedly at the door, either when close to it or from a distance	[48]
Behaviors oriented to the door (SE)	All active behaviors resulting in physical contact with the door, including scratching the door with the paws, jumping on the door, pulling on the door handle with the forelegs or mouth	[48,51]
Barking (SE)	Sharp explosive vocalization	[46,51]
Howling (SE)	Low-pitched, long-duration vocalization	[46,52]
Whining/Yelping (SE)	Whine: High-pitched vocalization Yelp: loud (relative to whining), high-pitched vocalization	[46]
Growling (SE)	Deep threatening rumble, with or without exposed teeth	[46]
Panting (SE)	Mouth open, tongue can be outside of mouth, quick and shallow breathing (inhalations/exhalations visible)	[53,54]
Paw lifting (PE)	A forepaw is lifted to a position of approximately 45°	[55]
Body shaking (SE)	The dog shakes his/her body	[46] *
Nose licking (SE)	Tongue extends upwards to cover nose, before retracting into mouth	[46,47]
Yawn (PE)	Mouth widely opened for a period of a few seconds, then closed	[46,47]
Tongue out (PE)	Licking the lips or the nose, and also keeping the tongue out even if it is not licking any part of the snout	[55,56,57]
OTHER BEHAVIORS	Any activity not included in the behavioral catalogue, such as self-grooming, digging, or circling	[46,55]

PE = point event, SE = state event. * = modified.

**Table 2:** Frequency and relative duration of presented behavior during 5 min of isolation.

OBSERVED BEHAVIORS				
Behavior	Frequency min-max	Median	Duration min-max (%)	Median (%)
Lying	0–2	1	0.0–81.5	51.5
Sitting	0–1	1	0.0–64.1	6.8
Standing	1–13	3.5	5.8–77.2	27.7
Exploration	1–4	3	0.8–10.4	1.8
Locomotion	0–13	2	0.0–72.0	3.2
Attention oriented to the door	2–24	13.5	5.3–78.7	46.0
Behaviors oriented to the door	0–2	0	0.0–1.1	0.0
Barking	0–3	0	0.0–0.6	0.0
Whining/Yelping	0–32	6.5	0.0–57.7	5.7
Body shaking	0–1	0	0.0–0.6	0.0

min-max = minimum-maximum

**Table 3:** Salivary ΔOXT (oxytocin (T1)–(T0)) and cortisol concentration during NC (negative condition, consisting of 5 min of isolation).

SALIVARY ΔOXT AND CORTISOL DURING NC						
Trainer	Dog № Sex		ΔOT (pg/mL)	CORTISOL (µg/dL)	ΔOXT Rank	CORTISOL Rank
A	1	F	77.5	0.233	8	8
A	2	F	15.85	0.116	4	4
B	3	M	20.77	0.164	5	6
B	4	M	−45.83	0.067	2	1
C	5	F	37.87	0.114	6	3
C	6	M	−31.86	0.129	3	5
D	7	M	66.36	0.176	7	7
D	8	F	−162.19	0.111	1	2

## References

[1] Kosfeld M., Heinrichs M., Zak P.J., Fischbacher U., Fehr E. (2005). Oxytocin increases trust in humans. Nature.

[2] Witt D.M., Sue Carter C., Walton D.M. (1990). Central and peripheral effects of oxytocin administration in prairie voles (Microtus ochrogaster). Pharmacol. Biochem. Behav..

[3] Insel T.R., Shapiro L.E. (1992). Oxytocin receptor distribution reflects social organization in monogamous and polygamous voles. Proc. Natl. Acad. Sci..

[4] Williams J.R., Insel T.R., Harbaugh C.R., Carter C.S. (1994). Oxytocin Administered Centrally Facilitates Formation of a Partner Preference in Female Prairie Voles (Microtus ochrogaster). J. Neuroendocrinol..

[5] Witt D.M. (1995). Oxytocin and rodent sociosexual responses: From behavior to gene expression. Neurosci. Biobehav. Rev..

[6] Insel T.R., Hulihan T.J. (1995). A gender-specific mechanism for pair bonding: Oxytocin and partner preference formation in monogamous voles. Behav. Neurosci..

[7] Olazabal D.E., Young L.J. (2006). Oxytocin receptors in the nucleus accumbens facilitate “spontaneous” maternal behavior in adult female prairie voles. Neuroscience.

[8] Ross H.E., Cole C.D., Smith Y., Neumann I.D., Landgraf R., Murphy A.Z., Young L.J. (2009). Characterization of the oxytocin system regulating affiliative behavior in female prairie voles. Neuroscience.

[9] Bosch O.J., Dabrowska J., Modi M.E., Johnson Z.V., Keebaugh A.C., Barrett C.E., Ahern T.H., Guo J., Grinevich V., Rainnie D.G. (2016). Oxytocin in the nucleus accumbens shell reverses CRFR2-evoked passive stress-coping after partner loss in monogamous male prairie voles. Psychoneuroendocrinology.

[10] Barrett C.E., Arambula S.E., Young L.J. (2015). The oxytocin system promotes resilience to the effects of neonatal isolation on adult social attachment in female prairie voles. Transl. Psychiatry.

[11] Tabbaa M., Paedae B., Liu Y., Wang Z. (2011). Neuropeptide regulation of social attachment: The prairie vole model. Compr. Physiol..

[12] Rincon A.V., Deschner T., Schülke O., Ostner J. (2020). Oxytocin increases after affiliative interactions in male Barbary macaques. Horm. Behav..

[13] Crockford C., Wittig R.M., Langergraber K., Ziegler T.E., Zuberbühler K., Deschner T. (2013). Urinary oxytocin and social bonding in related and unrelated wild chimpanzees. Proc. R. Soc. B Biol. Sci..

[14] Preis A., Samuni L., Mielke A., Deschner T., Crockford C., Wittig R.M. (2018). Urinary oxytocin levels in relation to post-conflict affiliations in wild male chimpanzees (Pan troglodytes verus). Horm. Behav..

[15] Benítez M.E., Sosnowski M.J., Tomeo O.B., Brosnan S.F. (2018). Urinary oxytocin in capuchin monkeys: Validation and the influence of social behavior. Am. J. Primatol..

[16] Wittig R.M., Crockford C., Deschner T., Langergraber K.E., Ziegler T.E., Zuberbühler K. (2014). Food sharing is linked to urinary oxytocin levels and bonding in related and unrelated wild chimpanzees. Proc. R. Soc. B Biol. Sci..

[17] Marotta R., Risoleo M.C., Messina G., Parisi L., Carotenuto M., Vetri L., Roccella M. (2020). The Neurochemistry of Autism. Brain Sci..

[18] Kong X.-J., Liu J., Li J., Kwong K., Koh M., Sukijthamapan P., Guo J.J., Sun Z.J., Song Y. (2020). Probiotics and oxytocin nasal spray as neuro-social-behavioral interventions for patients with autism spectrum disorders: A pilot randomized controlled trial protocol. Pilot Feasibility Stud..

[19] Gazzano A., Ogi A., Torracca B., Mariti C., Casini L. (2018). Plasma tryptophan/large neutral amino acids ratio in domestic dogs is affected by a single meal with high carbohydrates level. Animals.

[20] Marshall-Pescini S., Schaebs F.S., Gaugg A., Meinert A., Deschner T., Range F. (2019). The role of oxytocin in the dog–owner relationship. Animals.

[21] Kis A., Ciobica A., Topál J. (2017). The effect of oxytocin on human-directed social behaviour in dogs (Canis familiaris). Horm. Behav..

[22] Odendaal J.S.J., Meintjes R.A. (2003). Neurophysiological correlates of affiliative behaviour between humans and dogs. Vet. J..

[23] Mitsui S., Yamamoto M., Nagasawa M., Mogi K., Kikusui T., Ohtani N., Ohta M. (2011). Urinary oxytocin as a noninvasive biomarker of positive emotion in dogs. Horm. Behav..

[24] Romero T., Nagasawa M., Mogi K., Hasegawa T., Kikusui T. (2014). Oxytocin promotes social bonding in dogs. Proc. Natl. Acad. Sci. USA.

[25] Nagasawa M., Mitsui S., En S., Ohtani N., Ohta M., Sakuma Y., Onaka T., Mogi K., Kikusui T. (2015). Oxytocin-gaze positive loop and the coevolution of human-dog bonds. Science.

[26] Handlin L., Hydbring-Sandberg E., Nilsson A., Ejdebäck M., Jansson A., Uvnäs-Moberg K. (2011). Short-Term Interaction between Dogs and Their Owners: Effects on Oxytocin, Cortisol, Insulin and Heart Rate—An Exploratory Study. Anthrozoos.

[27] Handlin L., Nilsson A., Ejdebäck M., Hydbring-Sandberg E., Uvnäs-Moberg K. (2012). Associations between the psychological characteristics of the human–dog relationship and oxytocin and cortisol levels. Anthrozoos.

[28] Rehn T., Handlin L., Uvnäs-Moberg K., Keeling L.J. (2014). Dogs’ endocrine and behavioural responses at reunion are affected by how the human initiates contact. Physiol. Behav..

[29] MacLean E.L., Gesquiere L.R., Gee N.R., Levy K., Martin W.L., Carter C.S. (2017). Effects of affiliative human-animal interaction on dog salivary and plasma oxytocin and vasopressin. Front. Psychol..

[30] Jonas W., Johansson L.M., Nissen E., Ejdebäck M., Ransjö-Arvidson A.B., Uvnäs-Moberg K. (2009). Effects of intrapartum oxytocin administration and epidural analgesia on the concentration of plasma oxytocin and prolactin, in response to suckling during the second day postpartum. Breastfeed. Med..

[31] Brandtzaeg O.K., Johnsen E., Roberg-Larsen H., Seip K.F., MacLean E.L., Gesquiere L.R., Leknes S., Lundanes E., Wilson S.R. (2016). Proteomics tools reveal startlingly high amounts of oxytocin in plasma and serum. Sci. Rep..

[32] Uvnäs-Moberg K., Handlin L., Petersson M., Peggy McCardle James A., Griffin Valerie Maholmes S.M. (2011). Promises and pitfalls of hormone research in human-animal interaction. How Animals Affect Us: Examining the Influence of Human-Animal Interaction on Child Development and Human Health.

[33] MacLean E.L., Gesquiere L.R., Gee N., Levy K., Martin W.L., Carter C.S. (2018). Validation of salivary oxytocin and vasopressin as biomarkers in domestic dogs. J. Neurosci. Methods.

[34] Coulon M., Nowak R., Andanson S., Ravel C., Marnet P.G., Boissy A., Boivin X. (2013). Human-lamb bonding: Oxytocin, cortisol and behavioural responses of lambs to human contacts and social separation. Psychoneuroendocrinology.

[35] Onaka T., Takayanagi Y. (2019). Role of oxytocin in the control of stress and food intake. J. Neuroendocrinol..

[36] Buttner A.P. (2016). Neurobiological underpinnings of dogs’ human-like social competence: How interactions between stress response systems and oxytocin mediate dogs’ social skills. Neurosci. Biobehav. Rev..

[37] Marazziti D., Dell’Osso B., Baroni S., Mungai F., Catena M., Rucci P., Albanese F., Giannaccini G., Betti L., Fabbrini L. (2006). A relationship between oxytocin and anxiety of romantic attachment. Clin. Pract. Epidemiol. Ment. Heal..

[38] Tabak B.A., McCullough M.E., Szeto A., Mendez A.J., McCabe P.M. (2011). Oxytocin indexes relational distress following interpersonal harms in women. Psychoneuroendocrinology.

[39] Torres N., Martins D., Santos A.J., Prata D., Veríssimo M. (2018). How do hypothalamic nonapeptides shape youth’s sociality? A systematic review on oxytocin, vasopressin and human socio-emotional development. Neurosci. Biobehav. Rev..

[40] McQuaid R.J., McInnis O.A., Paric A., Al-Yawer F., Matheson K., Anisman H. (2016). Relations between plasma oxytocin and cortisol: The stress buffering role of social support. Neurobiol. Stress.

[41] Ng Z.Y., Pierce B.J., Otto C.M., Buechner-Maxwell V.A., Siracusa C., Werre S.R. (2014). The effect of dog–human interaction on cortisol and behavior in registered animal-assisted activity dogs. Appl. Anim. Behav. Sci..

[42] Buttner A.P., Thompson B., Strasser R., Santo J. (2015). Evidence for a synchronization of hormonal states between humans and dogs during competition. Physiol. Behav..

[43] Mariti C., Carlone B., Protti M., Diverio S., Gazzano A. (2018). Effects of petting before a brief separation from the owner on dog behavior and physiology: A pilot study. J. Vet. Behav..

[44] Mongillo P., Pitteri E., Carnier P., Gabai G., Adamelli S., Marinelli L. (2013). Does the attachment system towards owners change in aged dogs?. Physiol. Behav..

[45] Friard O., Gamba M. (2016). BORIS: A free, versatile open-source event-logging software for video/audio coding and live observations. Methods Ecol. Evol..

[46] Guardini G., Mariti C., Bowen J., Fatjó J., Ruzzante S., Martorell A., Sighieri C., Gazzano A. (2016). Influence of morning maternal care on the behavioural responses of 8-week-old Beagle puppies to new environmental and social stimuli. Appl. Anim. Behav. Sci..

[47] Tod E., Brander D., Waran N. (2005). Efficacy of dog appeasing pheromone in reducing stress and fear related behaviour in shelter dogs. Appl. Anim. Behav. Sci..

[48] Mariti C., Ricci E., Carlone B., Moore J.L., Sighieri C., Gazzano A. (2013). Dog attachment to man: A comparison between pet and working dogs. J. Vet. Behav. Clin. Appl. Res..

[49] Prato-Previde E., Custance D.M., Spiezio C., Sabatini F. (2003). Is the dog—Human relationship an attachment bond ? An observational study using Ainsworth’ s strange situation. Behaviour.

[50] Palestrini C., Previde E.P., Spiezio C., Verga M. (2005). Heart rate and behavioural responses of dogs in the Ainsworth’s Strange Situation: A pilot study. Appl. Anim. Behav. Sci..

[51] Mariti C., Carlone B., Ricci E., Sighieri C., Gazzano A. (2014). Intraspecific attachment in adult domestic dogs (Canis familiaris): Preliminary results. Appl. Anim. Behav. Sci..

[52] Parthasarathy V., Crowell-Davis S.L. (2006). Relationship between attachment to owners and separation anxiety in pet dogs (Canis lupus familiaris). J. Vet. Behav. Clin. Appl. Res..

[53] Dreschel N.A., Granger D.A. (2005). Physiological and behavioral reactivity to stress in thunderstorm-phobic dogs and their caregivers. Appl. Anim. Behav. Sci..

[54] Owczarczak-Garstecka S.C., Burman O.H.P. (2016). Can sleep and resting behaviours be used as indicators of welfare in shelter dogs (Canis lupus familiaris)?. PLoS One.

[55] Beerda B., Schilder M.B.H., van Hooff J.A.R.A., de Vries H.W., Mol J.A. (1998). Behavioural, saliva cortisol and heart rate responses to different types of stimuli in dogs. Appl. Anim. Behav. Sci..

[56] Rooney N., Gaines S., Hiby E. (2009). A practitioner’s guide to working dog welfare. J. Vet. Behav..

[57] Kotrschal K., Schöberl I., Bauer B., Thibeaut A.M., Wedl M. (2009). Dyadic relationships and operational performance of male and female owners and their male dogs. Behav. Processes.

[58] Cozzi A., Mariti C., Ogi A., Sighieri C., Gazzano A. (2016). Behavioral modification in sheltered dogs. Dog Behav..

[59] Scaglia E., Cannas S., Minero M., Frank D., Bassi A., Palestrini C. (2013). Video analysis of adult dogs when left home alone. J. Vet. Behav. Clin. Appl. Res..

[60] Cobb M.L., Iskandarani K., Chinchilli V.M., Dreschel N.A. (2016). A systematic review and meta-analysis of salivary cortisol measurement in domestic canines. Domest. Anim. Endocrinol..

[61] DeVries A.C., Glasper E.R., Detillion C.E. (2003). Social modulation of stress responses. Physiol. Behav..

[62] Petersson M., Uvnäs-Moberg K., Nilsson A., Gustafson L.L., Hydbring-Sandberg E., Handlin L. (2017). Oxytocin and cortisol levels in dog owners and their dogs are associated with behavioral patterns: An exploratory study. Front. Psychol..

[63] Lewandowski Jr G.W., Mattingly B.A., Pedreiro A. (2014). Under pressure: The effects of stress on positive and negative relationship behaviors. J. Soc. Psychol..

[64] Pirrone F., Ripamonti A., Garoni E.C., Stradiotti S., Albertini M. (2017). Measuring social synchrony and stress in the handler-dog dyad during animal-assisted activities: A pilot study. J. Vet. Behav. Clin. Appl. Res..

[65] Brown D., Anderson  R.K., Hart  B.L., Hart  L.A. (1984). Personality and gender influences on human relationships with horses and dogs. Pet Connection: Its Influence on Our Health and Quality of Life.

[66] Prato-Previde E., Fallani G., Valsecchi P. (2006). Gender differences in owners interacting with pet dogs: An observational study. Ethology.

[67] Javor A., Riedl R., Kindermann H., Brandstätter W., Ransmayr G., Gabriel M. (2014). Correlation of plasma and salivary oxytocin in healthy younq men - Experimental evidence. Neuroendocrinol. Lett..

[68] Grewen K.M., Davenport R.E., Light K.C. (2010). An investigation of plasma and salivary oxytocin responses in breast- and formula-feeding mothers of infants. Psychophysiology.

[69] Feldman R., Gordon I., Schneiderman I., Weisman O., Zagoory-Sharon O. (2010). Natural variations in maternal and paternal care are associated with systematic changes in oxytocin following parent-infant contact. Psychoneuroendocrinology.

[70] Feldman R., Gordon I., Zagoory-Sharon O. (2011). Maternal and paternal plasma, salivary, and urinary oxytocin and parent-infant synchrony: Considering stress and affiliation components of human bonding. Dev. Sci..

[71] Hernandez C.E., Thierfelder T., Svennersten-Sjaunja K., Berg C., Orihuela A., Lidfors L. (2014). Time lag between peak concentrations of plasma and salivary cortisol following a stressful procedure in dairy cattle. Acta Vet. Scand..

[72] Cimarelli G., Turcsán B., Bánlaki Z., Range F., Virányi Z. (2016). Dog Owners’ Interaction Styles: Their Components and Associations with Reactions of Pet Dogs to a Social Threat. Front. Psychol..

